# Role of intra-operative contrast-enhanced ultrasound (CEUS) in robotic-assisted nephron-sparing surgery

**DOI:** 10.1007/s11701-015-0496-1

**Published:** 2015-02-07

**Authors:** Ahmad N. Alenezi, Omer Karim

**Affiliations:** 1 Department of Urology, Frimley Health NHS Foundation Trust, Wexham Park Hospital, Slough, Berkshire, SL2 4HL UK; 2 Minimal Invasive and Robotic Surgery Fellow, Frimley Health NHS Foundation Trust, Wexham Park Hospital, Wexham, SL2 4HL Berkshire, UK; 3 Consultant Urological Surgeon, Frimley Health NHS Foundation Trust, Wexham Park Hospital, Wexham, SL2 4HL Berkshire, UK

**Keywords:** Robotic assisted, Partial nephrectomy, Nephron sparing, Zero-ischemia, CEUS, Contrast-enhanced ultrasound scan, SonoVue

## Abstract

This review examines studies of intra-operative contrast-enhanced ultrasound (CEUS) and its emerging role and advantages in robotic-assisted nephron-sparing surgery. Contrast-enhanced ultrasound is a technology that combines the use of second-generation contrast agents consisting of microbubbles with existent ultrasound techniques. Until now, this novel technology has aided surgeons with procedures involving the liver. However, with recent advances in the CEUS technique and the introduction of robotics in nephron-sparing surgery, CEUS has proven to be efficacious in answering several clinical questions with respect to the kidneys. In addition, the introduction of the microbubble-based contrast agents has increased the image quality and signal uptake by the ultrasound probe. This has led to better, enhanced scanning of the macro and microvasculature of the kidneys, making CEUS a powerful diagnostic modality. This imaging method is capable of further lowering the learning curve and warm ischemia time (WIT) during robotic-assisted nephron-sparing surgery, with its increased level of capillary perfusion and imaging. CEUS has the potential to increase the sensitivity and specificity of intra-operative images, and can significantly improve the outcome of robotic-assisted nephron-sparing surgery by increasing the precision and diagnostic insight of the surgeon. The purpose of this article is to review the practical and potential uses of CEUS as an intra-operative imaging technique during robotic-assisted nephron-sparing surgery.

## Introduction

The therapeutic management of renal cell carcinoma is mainly surgical. In this regard, the safe, efficient, and minimally invasive technique of nephron-sparing surgery or partial nephrectomy (PN) has gained popularity in the past decade, concurrent with advances in modern imaging and surgical techniques.

In select patients with renal tumors less than 4 cms in size, partial nephrectomy is the recommended option [1, 2]. Partial nephrectomy enables preservation of renal function, by removing renal tissue limited to the tumor and immediate periphery only. Although the older laparoscopic partial nephrectomy technique (LPN) is more widely used, robotic-assisted partial nephrectomy (RPN) has gained prominence recently, as RPN is technically more advantageous with a shorter learning curve [3] and a shorter warm ischemia time (WIT) [4] as compared to LPN.

Since a prolonged warm ischemic time can impair renal function or aggravate chronic renal impairment, zero-ischemia or selective ischemia PN, performed by ligating or clamping selected arteries is gaining prominence over the long-established hilar vessel clamping technique. WIT is reduced considerably, with the avoidance of global ischemia of the kidney by clamping selected arteries supplying blood to the segment of the kidney that contains the tumor.

In addition, intra-operative ultrasonography is very useful during partial nephrectomy, as it assists complete tumor resection with improved visualization of the margins of the tumor. Although, the Doppler probe, FireFly fluorescence imaging with indocyanine green (ICG), and drop-in ultrasound with power Doppler are intra-operative techniques presently in use to help facilitate zero-ischemia PN [5], these techniques have certain limitations. On the other hand, a contrast-enhanced ultrasound (CEUS) can enhance the visualization of the tumor and its vascularization during a RPN with more precision consequently increasing the diagnostic acumen of the surgeon. The purpose of this article is to review the practical and potential uses of CEUS as an intra-operative imaging technique during robotic-assisted nephron-sparing surgery.

## Review of studies on CEUS in RPN

We performed a comprehensive literature search by electronic bibliographic databases in MEDLINE, Cochrane databases and PubMed up to July 2014 using the following keywords: “contrast enhanced ultrasound”, “intra-operative ultrasound”, “nephron-sparing surgery”, “partial nephrectomy” and “robotic-assisted partial nephrectomy”. The list of all electronically identified bibliographies and articles was then reviewed to distinguish potentially relevant studies including experiments, case reports, and reviews and preliminary clinical studies. In addition, the supplements of major journals were hand-searched to identify relevant abstracts that had not been published as peer-reviewed articles. Finally, we selected studies in the field of intra-operative ultrasound in laparoscopic and robotic-assisted partial nephrectomy. There were five relevant studies [5, 6, 9, 12, 13]. The largest population among the relevant studies had a population size of 22 patients [9].

## Methods and techniques

Contrast-enhanced ultrasound or CEUS is a novel intra-operative imaging technique that combines the microbubble technology of contrast media with complementary ultrasound technology. The contrast agent used during the CEUS procedure enhances the kidneys for about 2 min in real-time post-injection of contrast. However, in the presence of underlying chronic kidney disease, the contrast enhancement of the renal parenchyma remains for a shorter period and is not as intense as seen with a normally functioning kidney [6].

A second-generation contrast agent, SonoVue (Braco, Milan, Italy) is widely used for the CEUS procedure. Each milliliter of this contrast agent contains 8 µL of stabilized microbubbles of sulfur hexachloride gas [7]. The recommended dose for renal imaging using a single intravenous injection of SonoVue is 1–2.4 ml. If the kidneys are imaged in contrast-enhanced mode from before the injection of SonoVue, the renal parenchyma appears dark and the ultrasound contrast agent can actually be seen to flow into the renal parenchyma usually within 15–20 s after an intravenous injection of SonoVue [5]. This contrast enhancement of the renal parenchyma starts with the medulla and spreads to the renal cortex as the kidney is perfused with ultrasound contrast agent. Intravenous aliquots of SonoVue may be repeated as necessary and most importantly, this contrast agent is not nephrotoxic, as it is excreted by the lungs. Thus, it can be used safely in patients with compromised renal functions [8].

Different surgeons use different techniques to perform RPN, depending on tumor characteristics, patient factors, surgical experience and available technology. Kaczmarek et al. [9] performed RPN using a robotic US probe for tumor identification in 22 patients. The Gerota’s fascia was opened to expose the renal capsule around the tumor, hilar blood vessels were then clamped in preparation for excision of the tumor under warm ischemia and the sliding clip renorrhaphy technique was used for renal reconstruction [10]. The ultrasound probe was introduced through the assistant port to obtain images to enable the recognition of the junction between the tumor and normal renal parenchyma. The location and extent of the tumor were visualized through the medium of real-time images, obtained from intra-operative ultrasound techniques. Images were produced and visualized by the surgeon using the TilePro feature of the da Vinci surgical system to produce a picture-on-picture image in the console screen to view the images [11]. The margins of resection were then marked with cautery to include a rim of normal renal parenchyma and tumor excised around this margin (Tables 1, 2).

**Table 1:** The advantages and limitations of different intraoperative imaging techniques for robot-assisted nephron-sparing surgery

	Power doppler	ICG	CEUS
Segmental blood flow	✓	✓	✓
Medullary blood flow	✓	✗	✓
Nondefatted kidney	✓	✗	✓
No toxicity	✓	?	✓
No artifact with probe movement	✗	✓	✓

Amrith Raj Rao, Robert Gray, Erik Mayer, Hanif Motiwala, Marc Laniado, Omer Karim, “Occlusion Angiography Using Intraoperative Contrast-enhanced Ultrasound Scan (CEUS): a novel technique demonstrating segmental renal blood supply to assist zero-ischemia robot-assisted partial nephrectomy”. Eur Urol 2013; 63:913–919

*ICG *Indigo green, *CEUS* Contrast enhanced ultrasound

**Table 2:** List of contrast agents available in practice

Name	Shell composition	Gas	Manufacturer
AI-700	Polymer	Perfluorocarbon	Acusphere
biSphere	Gelatin/polymer	Air	Point Biomedical
BR14	Phospholipid	Perfluorobutane	Bracco Diagnostics
BY 963	Lipid	Air	Byk-Gulden
Levovist	Galactose/palmitic acid	Air	Schering
Definity	Lipid	Perfluoropropane	Bristol-Myers Squibb
Imagent	Surfactant	Perfluorocarbon	Imcor Pharmaceuticals
Optison	Albumin	Perfluoropropane	GE Healthcare
Sonazoid	Lipid	Perfluorobutane	GE Healthcare
SonoVue	Surfactant	SF6	Bracco Diagnostics
MRX-408	Lipid/ligand oligopeptide	Perfluoropropane	ImaRx
Quantison	Albumin	Air	Andaris Ltd
QFX	Albumin	Perfluorocarbon	Guangzhou Nanfang Hospital

Ji-Bin Liu, Gervais Wansaicheong, Daniel A. Merton, Flemming Forsberg, Barry B. Goldberg, “Contrast-enhanced Ultrasound Imaging: State of the Art”, J Med Ultrasound 2005;13(3):109–126

In essence, the intra-operative CEUS technique uses two images: (1) a conventional B-mode or 2D mode (brightness mode) image of the tissue using low acoustic power that produces a two-dimensional image on the screen and (2) a contrast-enhanced mode (a contrast-specific) image which displays the reflection made by the spatial distribution of bubbles.

Microbubbles vibrate under the pressure changes induced by the probe transmitter. This oscillation produces energy that are detected by the transducer and converted into an image. Microbubbles are capable of withstanding inertial cavitation. However, higher ultrasonic transmission power causes destruction of the microbubbles and adversely affects the signals from the target. The contrast-enhanced mode uses a lower transmission than that for the non-contrast imaging to avoid destruction of the microbubble and minimize the transmitted signals from the tissues, and obtain a real time, a series of contrast-specific images.

**Fig. 1:**
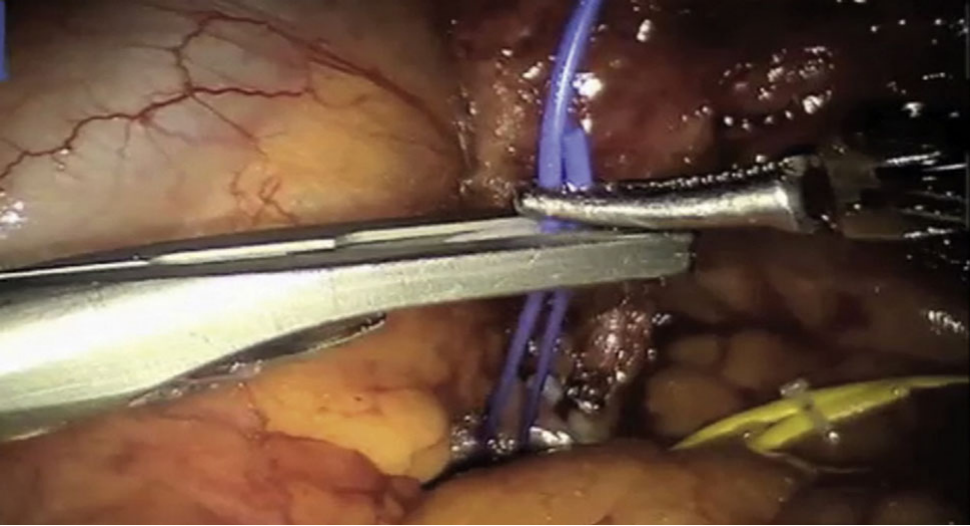
Branches of the renal artery identified with different colored vessel loops for improved identification [5]

In our institution we previously described a technique combining intra-operative CEUS and selective occlusion angiography [5]. Using the fourth-arm, transperitoneal approach on 5 consecutive patients undergoing RPN for clinically T1 renal cell carcinoma. A 5-mm assistant port was utilized for suction and retraction of the bowel, and a 12-mm assistant port to insert the drop-in US probe, as well as for the application of the vascular bulldog clamps, and the Hem-o-lock clips. After mobilization of the overlying bowel loops, hilar dissection was performed to separate the renal artery branches. The branches were individually color-coded with vessel loops as illustrated in Fig. 1. The tumor was then identified with an intra-operative ultrasound scan using a ProART robotic drop-in probe. The Gerota’s fascia opened some distance away from the tumor, followed by dissection of fat and fascia towards the tumor. After adequate dissection was achieved, the ultrasound drop-in probe was reinserted to scan and assess the tumor position and intraparenchymal depth. As a novel technique occlusion angiography was then performed, by selective clamping of the arterial branches to obtain devascularization of a discrete area around the tumor. Once the arterial clamping was complete, 1 ml of SonoVue ultrasound contrast agent was injected intravenously. An ultrasound scan in contrast-enhanced ultrasound mode was performed to monitor the circulation in the kidneys and segment containing the tumor.

**Fig. 2:**
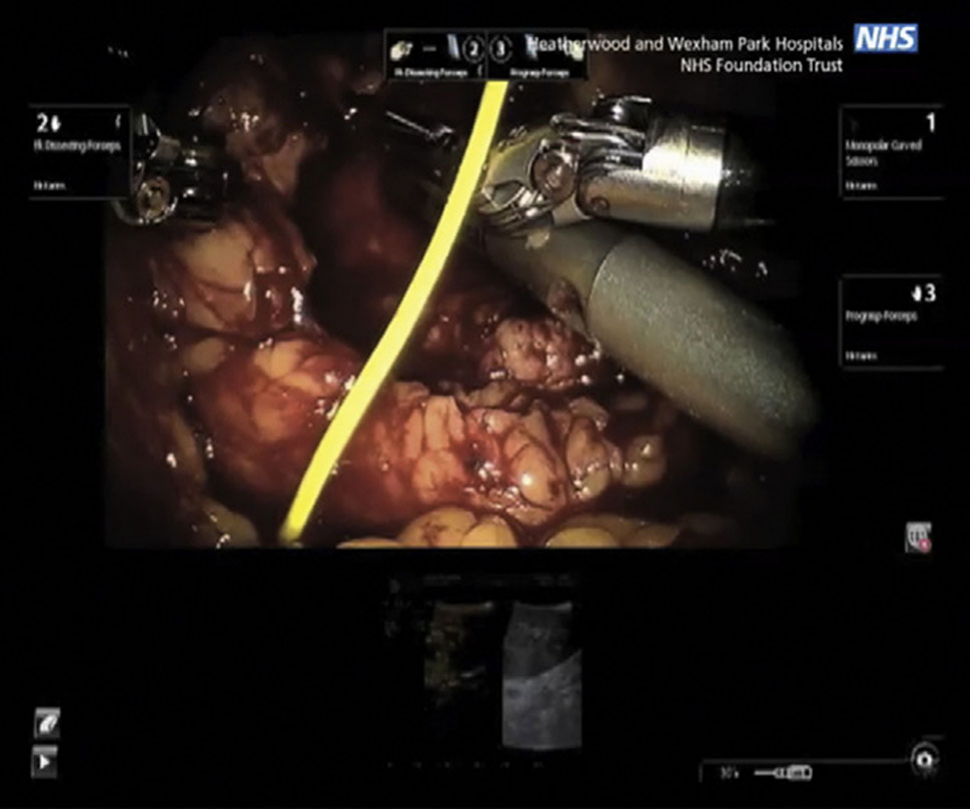
Using the robotic ProGrasp forceps the surgeon manipulates the robotic ultrasound probe over the kidney, to visualize the position of the tumor (B-mode ultrasound), and assess blood flow (using CEUS). The operating surgeon views the ultrasound images in the TilePro mode on the robot console. The B-mode ultrasound image is on the right, while the CEUS image is on the left of the TilePro image [5]

**Fig. 3:**
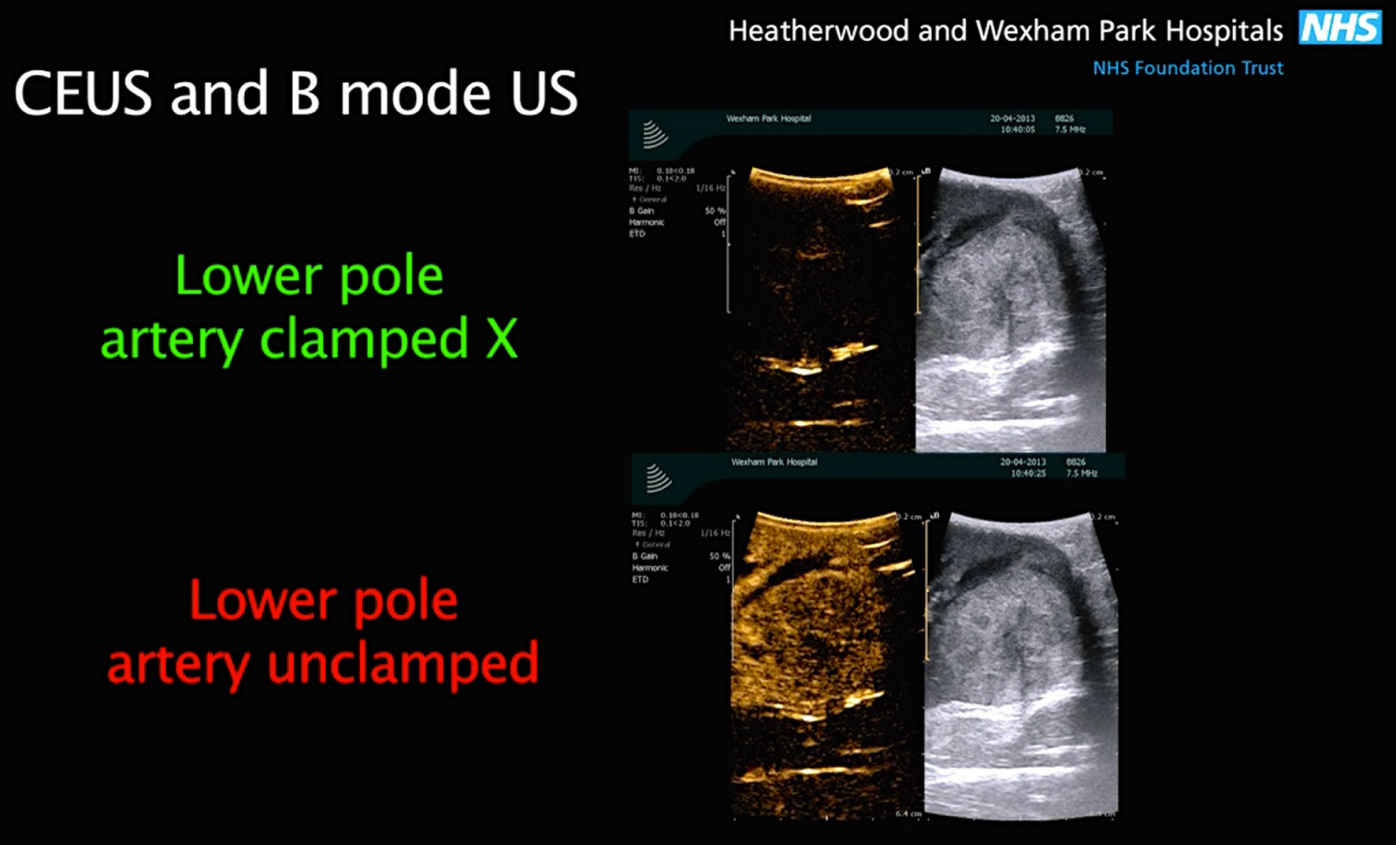
the difference between a conventional B-mode images on the right and the contrast-enhanced mode images on the left. SonoVue microbubble contrast agent was injected intravenously. The contrast-enhanced ultrasound mode in the monitor shows the circulation in the kidney and the segment containing the tumor in the lower pole. This facilitates and confirms selective ischemia to the desired renal segment

The contrast-enhanced ultrasound mode was used to avoid destruction of the microbubbles (vide infra). The TilePro mode was then selected to observe these images on the robotic console as seen in Figs. 2, 3. The cold-cut scissors and ProGrasp forceps (Intuitive Surgical) were then utilized for tumor excision, following which the renal cortex was closed using the sliding clip renorrhaphy technique.

**Fig. 4:**
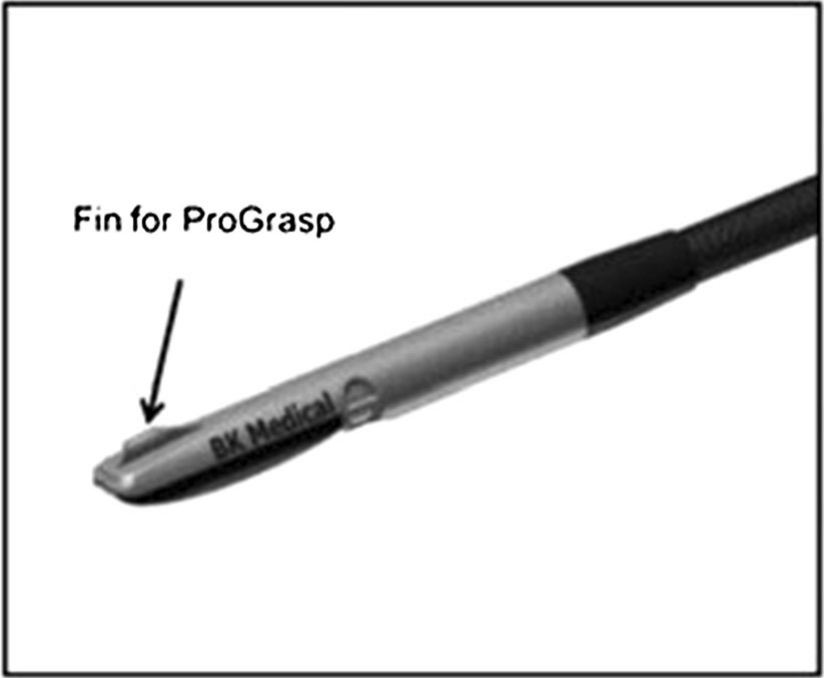
The ProART™ Robotic Transducer Type 8826 (courtesy of BK Medical)

RPN generally involves the handling and control of the laparoscopic ultrasound probe by the assistant; hence, limiting the autonomy and precision of the surgeon. This is where the novel technology of the ProART robotic transducer can play an important role (Fig.  4).

Using a ProART robotic transducer 8826 (BK Medical) for the purpose of intra-operative ultrasonography increases the acumen and precision of the surgeon [9]. It has a curved linear array, with a large field of view, and a 12-5 MHz transducer resolution. It also provides unique 3D visualization, and excellent fingertip control, as the fin is located directly over the transducer array.

Further more, this probe fits through a standard trocar and provides a sector 36° image field. This probe facilitates accurate imaging with maximum control in the hands of the surgeon, since its fin is located over the transducer array and the ProGrasp forceps can pick the fin easily providing improved control.

**Fig. 5:**
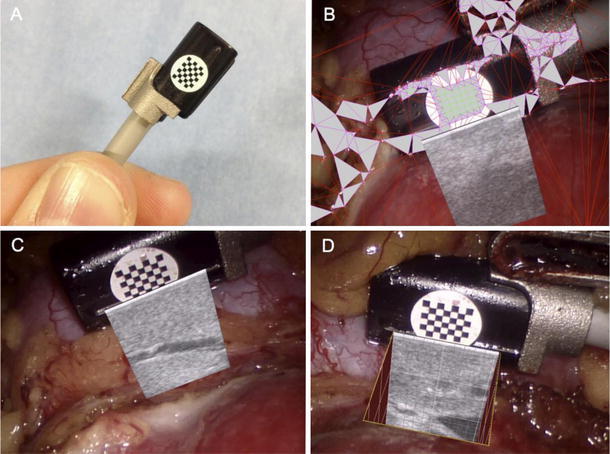
**a** Ultrasound probe with attached chessboard pattern mounted in custom-made clip; **b** real-time automatic tracking and registration process; **c** superimposed ultrasound; **d** superimposed ultrasound with cutaway and 1-mm ruler (courtesy of Mayer et al.)

One of the failures of current intra-operative ultrasound technology is the requirement of the surgeon to correlate the real-time subsurface ultrasound image to the separate console intra-operative robotic view. In this regard Mayer E et al. [12] recently reported preliminary result of a novel method using live registered intra-operative ultrasound overlay but without the utilization of CEUS. This method of live image registration involved a three-step process of calibration, image registration, and finally image overlay, and has shown a registration accuracy <0.5 mm. The real-time ultrasound image registration was attained using a stepwise process. First, prominent features were identified in the intra-operative endoscopic image. Second, irregular and outsized triangles were discarded. Third, using the camera calibration, transformation to camera coordinate systems was determined. Fourth, the result was concatenated with ultrasound image-to-probe transformation determining where to render the ultrasound image in the intra-operative endoscopic views. Finally, the live ultrasound image was superimposed on the stereo console display. The ultrasound image was available for display in two different viewing styles. The first was a simple overlay with variable transparency (allowing the surgeon to see the tissue underlying the ultrasound view); the second enabled a ‘‘cutaway’’ feature. Within this cutaway view the image was displayed as the posterior aspect of a cube, allowing the surgeon to appreciate depth more easily [12] Fig. 5.

The authors reported some limitations. The method reported delay in video processing which currently necessitated the two views to be displayed simultaneously. More importantly further work still needed to delineate the blood supply of the tumor in relation to its location.

In our views, incorporating CEUS with selective renal occlusion angiography may solve some of those limitations. Thereby, further research is required to evaluate the role of those techniques in improving tumor resection, clinical outcomes and long-term renal function.

## Discussion

Until recently, the use of CEUS has been limited to its clinical applications on the liver; however, due to its safety and cost-effectiveness, it has gained importance answering several clinical questions related to the kidneys. In select cases, CEUS can also negate the need for magnetic image resonance and computed tomography [13].

Contrast-enhanced ultrasound is particularly useful in imaging the kidneys. CEUS can differentiate solid renal tumors from pseudo-tumors and cystic lesions in the kidneys. It has also shown advantages in characterization of renal lesions and also by being able to differentiate between a non-perfused kidney lesion and normal perfused renal tissue. In addition, CEUS can help classify complex cystic renal masses according to the Bosniak classification system. Visualization of renal trauma, ischemia, and infections can also be demonstrated. CEUS has also been utilized in vascular imaging for renal artery stenosis, and appraisal of percutaneous ablation therapy for renal tumors. These indications for the use of CEUS have been set by The European Federation of Societies indications for CEUS for Ultrasound in Medicine and Biology (EFSUMB) in its 2011 updated Guidelines and Recommendations [6].

Intra-operative technology currently used for real-time imaging of the renal blood flow is mainly FireFly fluorescence with indocyanine green (ICG), and the drop-in ultrasound with power Doppler. Regrettably, these techniques have some limitations. Power Doppler is dependent on the movement of blood in vessels for real-time imaging of renal vasculature. Consequently, accidental movement of the probe over the surface of the kidney can give a false positive artifact by mimicking blood flow within the kidney. In practice, power Doppler may, thus, only be more useful over the renal hilum where the vessels are much larger and minor movement artifacts relatively less important. CEUS is not affected by movement artifact of the ultrasound probe and which makes CEUS a better technique in comparison to the power Doppler [5].

Another limitation of Firefly is that it only allows visualization of blood flow on the surface of the kidney devoid of perinephric fat or skeletonized vessels [5]. Removal of perinephric fat in patients with sticky or toxic fat can also increase the time taken to complete a RPN procedure. Defatting a kidney to visualize the renal cortex could theoretically compromise oncological margins in a patient with microscopic pT3a disease. It is fortunate that, CEUS overcomes the limitations of the Firefly system, by enabling visualization of the renal blood flow and the location of tumor through the perinephric fat [5].

Firefly fluorescence imaging is used in conjunction with the da Vinci Si surgical system. At the time of writing, it has not yet been approved for use with the latest da Vinci Xi model. Although, Firefly technology enables real-time imaging of renal perfusion with identification of important anatomical detail, it requires a special camera, light source and telescope to detect the florescence, which makes this technique more expensive.

There is a growing interest to minimize costs, improve quality and optimize access to new health care technologies. Similarly, many of the new and or evolving technologies can only be effectively evaluated after widespread use in clinical practice.

RPN using either ICG or CEUS technology, essentially requires a robotic intra-operative US transducer probe e.g. ProART robotic transducer 8826 (BK Medical). The estimate cost is in the range of $ 20,000–23,000 (estimate supplied by the UK distributor).

Compared to ICG, CEUS requires an additional estimate cost (supplied by U.K distributor) in the range of $ 3,000–5,000 for the integrated CEUS software (BK Medical) and $ 70–80 per vial cost of contrast agent including cannulation e.g. SonoVue (Bracco Diagnostics).

**Fig. 6:**
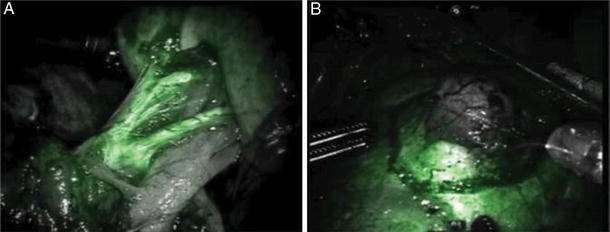
Fluorescence imaging during robotic partial nephrectomy using intravenously injected ICG illustrating on the *left*: **a** arterial phase enhancement of the primary, secondary, and tertiary arterial branches of the kidney and on the right: **b** hypo-fluorescent renal mass with surrounding normal fluorescent renal parenchyma (Courtesy of Silvers et al.) [15]

In contrary, ICG has extra cost of $100,000 for the near infrared fluorescence imaging (NIRF) integrated robotic camera system and $100 per vial cost of ICG [14]. Figure 6

A fact worth noting, ICG used in Firefly technique contains sodium iodide, which could potentially have a risk of anaphylactic shock. It is therefore important to check a patient’s past history of allergy prior to administering ICG. On the other spectrum, despite a minor concern that interaction between ultrasound and the microbubble contrast agents may lead to theoretical in vitro hemolysis and cell death at capillary levels. However, extensive studies ostensibly demonstrated that those concerns have not been clinically encountered. Similarly safety analysis of SonoVue in more than 20,000 patients revealed a rate of serious adverse effects of 0.0086 % [16–20].

SonoVue comprises of gas microbubbles similar in size to red blood cells. Microbubble contrast agents circulate for several minutes inside the blood vessels lumen then they dissolve. Each of these microbubbles is covered outwardly by a lipid, protein, or polymer coating shell. The lungs excrete the gas contained in the microbubbles, while the liver metabolizes the protein, lipid, or polymer shell [8].

SonoVue a second-generation ultrasound contrast agent used in the CEUS procedure is non-allergenic and does not interfere with renal function, as it is not excreted by the kidneys, unlike the contrast agents used in other imaging techniques. It is, therefore, not contra-indicated in patients with impaired renal function.

Studies have confirmed that the kidney and the pelvicalyceal system have no role in the accumulation and excretion or of the microbubble contrast agents. More over, recent reports have demonstrated a valuable a supportive diagnostic role of CEUS in acute and chronic rejection after renal transplantation [21, 22].

Due to of this metabolic pathway, renal impairment is not a contraindication for the use of microbubble contrast agents. Hence in conditions of reduced renal blood perfusion, ischemia, and diabetic nephropathy; the short duration of uptake of the microbubble contrast agent can safely be overcome by administering the agent as multiple injections [23].

When an ultrasound wave falls on the microbubbles, they expand to almost double their original size and contract simultaneously, producing an oscillatory movement. This movement further results in the transmission of return signals to the US machine transducer [24], resulting in successful enhancement of the renal microvasculature and accurate tumor marking. A technique, which we are still developing, is sequential occlusion angiography. In this technique we capitalize on the ability to rapidly destroy or “rupture” the SonoVue microbubbles by increasing the ultrasound scanning frequency. This effectively clears the renal parenchyma or tissue being scanned of microbubbles and allows a second or subsequent intravenous injection of SonoVue to be administered immediately. In our hands, this is the real advantage of CEUS, which undoubtedly, seems to offer a better intra-operative imaging in comparison to power Doppler and Firefly.

The combination of CEUS and microbubble contrast agents allows a definite enhancement of contrast resolution, and inhibition of signals from stationary tissues. Although, SonoVue is more widely used for CEUS in most countries except the U.S., there are a number of other alternative contrast agents available for this purpose.

In recent years, there has been a positive shift towards robotic-assisted partial nephrectomy in comparison to laparoscopic partial nephrectomy, due to its ability to reduce the WIT and learning curve during nephron-sparing surgery [3].

The robotic technique of partial nephrectomy was first performed, and subsequently published by Gettman and colleagues in 2004 [25]. A prolonged WIT is potentially deleterious to the recovery of the renal functions post RPN, especially in patients with high risk factors, or underlying disorders such as hypertension, diabetes, and small vessel disease [26, 27]. Surgeons at present are encouraged to avoid global ischemia and consequently reduce the WIT. Ligating or clamping selective arteries that supply blood to the segment of the kidney containing the tumor helps achieve a lower WIT.

Intra-operative ultrasound is invaluable for this purpose, as it can demonstrate real-time imaging of the renal vasculature. CEUS is capable of further reducing the WIT by aiding the process of selective clamping, since it permits real-time scanning of the macrovasculature and microvasculature of the kidneys without the need for removing the perinephric fat.

**Fig. 7:**
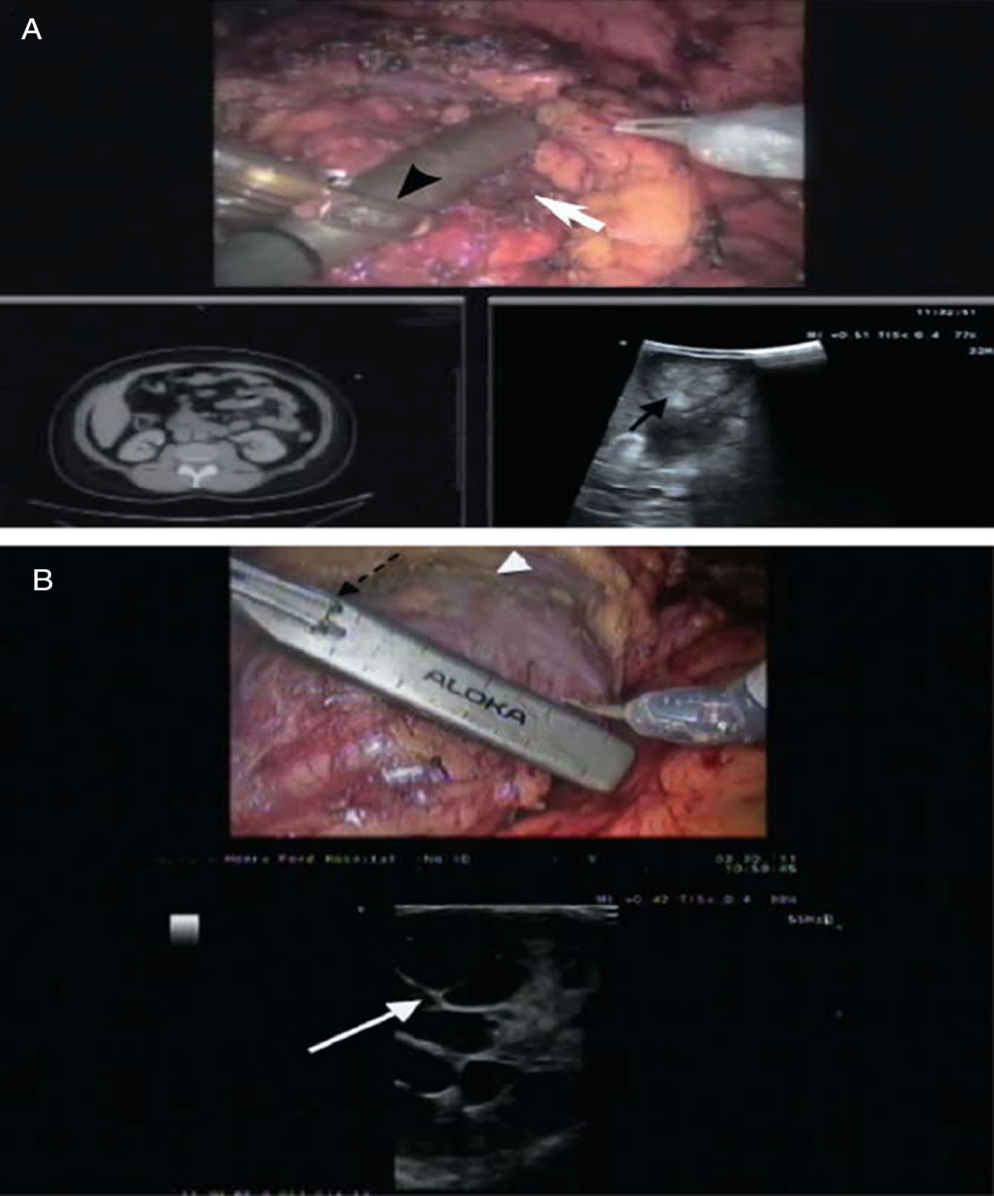
**a** Laparoscopic ultrasound probe being used for a right renal mass identification (a solid *white arrow* on the upper console image; solid *black arrow* on lower TilePro ultrasound image). The surgeon is trying to grab the laparoscopic probe with the robotic instrument to adjust the position (*black arrow-head*). **b** Robotic ultrasound probe being used to identify a right renal cystic renal cell carcinoma (*solid white arrow*). The robotic instrument is engaged with the notch on the probe (*dashed black arrow*), allowing the surgeon to independently maneuver the probe to identify tumor margins. The *arrow head* notes the scored resection margin of the far side of the tumor [8]

Intra-operative imaging using a robotic ultrasound probe can significantly increase the diagnostic acumen of the surgeon, with its high-resolution real-time images, which may thus improve outcomes of RPN in patients post-surgery. Laparoscopic ultrasound probes for intra-operative scanning have limitations and may reduce surgical precision, as the assistant holds and manipulates the laparoscopic ultrasound probe. The laparoscopic ultrasound probe is also prone to slipping off the kidney surface, and requires the assistance of a robotic instrument for repositioning the probe or to prevent it slipping off the kidney [9]. A robotic ultrasound probe gives the surgeon full autonomy in the surgical field, as the fin is placed just over the transducer array and is controlled by the surgeon. The robotic ultrasound probe also eliminates the issue of instrument clashing in the operating field [9]. Figure 7 shows the difference between a laparoscopic US probe and a robotic US probe.

CEUS performed with a robotic ultrasound probe, thus, aids improved identification of the tumor, mapping of the renal blood vessels, and precise resection of the tumor. Tumors generally have good vascularity, a feature which can enhance the quality of the signals detected with CEUS and the robotic probe. In our experience, when combining selective occlusion angiography with CEUS using the robotic ProART probe, CEUS was more useful in assessing the regions of ischemia and perfusion, in comparison to power Doppler.

Contrast-enhanced ultrasound does have some limitations. Although they are few in number and its benefits outweigh them. The contrast agents used in the CEUS imaging are not nephrotoxic, but they are contra-indicated in patients with underlying cardiopulmonary disorders, since the lungs and liver excrete the microbubbles [27, 28]. Also, due to the limited period of tissue enhancement, which is dependent on perfusion with the microbubble contrast agents, following injection of ultrasound contrast agent, CEUS can visualize only one kidney at a time, unlike Magnetic resonance imaging and computed tomography, in which both kidneys are scanned at the same time.

## Conclusion

Contrast-enhanced ultrasound has a wide range of applications for diagnostic imaging and more recently real-time intra-operative surgical imaging. Although, more widely used in relation to the liver, it could be very effective in addressing a number of clinical issues with respect to the kidneys. Used with the microbubble contrast agents, it can facilitate imaging of renal tumors during RPN, especially in patients with impaired renal function who cannot be given the other contrast agents used in current imaging techniques. CEUS can successfully reduce global WIT and thus may improve recovery of renal function. By facilitating selective arterial clamping during RPN and avoiding global ischemia it may decrease the risk of permanent loss of nephrons. Most importantly, CEUS can help us image the renal microvasculature, without affecting renal function. In addition, CEUS is capable of dynamic evaluation and quantification of microvasculature blood (capillary perfusion) in real time. When used in conjunction with a robotic ultrasound probe, CEUS can facilitate better visualization of renal vasculature and tumor and ultimately improving acumen and precision. CEUS is a valuable and a cost-effective tool for identification of renal blood flow in RPN, especially with complex, challenging tumors.
